# Prenatal Low-Protein and Low-Calorie Diets Differentially Alter Arcuate Nucleus Morphology in Newborn Male Rats

**DOI:** 10.3389/fnana.2022.896732

**Published:** 2022-06-16

**Authors:** Noemí Blanco, Jose Manuel Fernández-García, Beatriz Carrillo, Antonio Ballesta, Rocío García-Úbeda, Paloma Collado, Helena Pinos

**Affiliations:** ^1^Department of Psychobiology, National University of Distance Education, Madrid, Spain; ^2^University Institute of Research-UNED-Institute of Health Carlos III (IMIENS), Madrid, Spain; ^3^Faculty of Psychology, Universidad Villanueva Madrid, Madrid, Spain; ^4^Department of Psychology, Faculty of Biomedical and Health Sciences, Universidad Europea de Madrid, Madrid, Spain

**Keywords:** low-protein and caloric diet, gestational exposure, arcuate nucleus (ARC), hypothalamus, sex differences

## Abstract

**Background:**

Malnutrition during the early stages of development produces alterations that can compromise the functioning of the hypothalamic circuits that regulate food intake. The purpose of this study is to analyze the effects that a low-protein and low-calorie diet has on the morphology of the arcuate nucleus (ARC) of the hypothalamus in newborn male and female rats.

**Methods:**

On gestational day 6 (G6), six pregnant rats were divided into two groups. One group was made up of three pregnant rats, which were fed *ad libitum* with a control diet (20% casein), and the other one was made up of three pregnant rats, which were fed *ad libitum* with a low-protein diet (8% casein) and 30% of a calorie-restricted diet. On the day of birth, pups were sacrificed, resulting in four experimental groups: control male, control female, low-protein and low-calorie diet male, and low-protein and low-calorie diet female (*n* = 5 in each group). The volume and number of neurons, together with the neuronal density and number of apoptotic cells, were measured.

**Results:**

Males on a low-protein and low-calorie diet showed a significant increase in the number of neurons and in the neuronal density of the ARC with regard to the rest of the groups studied. These increases were also reflected in the posterior part of the nucleus. Although the existence of sexual dimorphism was not detected in any of the parameters studied in the control groups, the number of neurons and neuronal density showed differences between males and females fed with a low-protein and low-calorie diets due to the increase in the number of neurons shown by the male. No significant differences were found in the number of apoptotic cells.

**Conclusion:**

Our results show that a low-protein and low-calorie diet during the prenatal stage produces alterations in the ARC of the hypothalamus in newborn animals and, more importantly, that the effects of malnutrition are evident in males but not in females. Therefore, it is essential to follow a balanced diet during the early stages of life to ensure optimal development of the neural circuits that regulate eating.

## Introduction

There is conclusive evidence that the period of development is crucial for the programming of an adequate energy metabolism and the brain circuits that regulate it (Dearden and Ozanne, 2015; Pinos et al., 2018). In this programming, a balanced diet plays a crucial role since overnutrition or dietary restriction can seriously compromise the regulation of homeostasis. High-fat diets during prenatal stages increase the risk of metabolic syndrome-related disorders such as diabetes, obesity, or heart diseases (Desai et al., 2016; Paradis et al., 2017; Preston et al., 2020; Rodgers and Sferruzzi-Perri, 2021) and, besides, they alter the brain morphology (Argente-Arizón et al., 2016; Carrillo et al., 2016). Likewise, severe dietary restrictions result in body weight loss together with both metabolic and brain alterations (Bedi, 1991; Plagemann et al., 2000; Pinos et al., 2004, 2011; Rocha et al., 2014; Carrillo et al., 2016), or may also result in disorders, which are associated with metabolic syndrome, whether high-fat or high-sugar diets are available later in life (Vickers et al., 2000, 2005). The onset of all these disorders is mainly due to the malnutrition-induced alterations in the morphology and physiology of the circuits that control food intake (Taylor and Poston, 2007; Desai et al., 2015; Rivera et al., 2019; Sertie et al., 2020).

The arcuate nucleus (ARC) is a hypothalamic structure strategically located at the base of the third ventricle, close to the median eminence, which receives direct input of the body’s metabolic status from the digestive system and adipose tissue. In rodents, the hypothalamus develops in the late embryonic and early fetal periods (Rice and Barone, 2000). Specifically, the period of neurogenesis in the ARC occurs between embryonic days (E) 11 and E16, and the primary connections from the ARC to hypothalamic nuclei involved in the regulation of energy metabolism, such as lateral (LH), dorsomedial (DMH), ventromedial (VMH), and paraventricular (PVH) hypothalamus occurs mostly during the early postnatal period until postnatal day (P) 18, as their connections reach the adult pattern by P12 (Bouret et al., 2004; Padilla et al., 2010; Ishii and Bouret, 2012). Along with neurogenesis, many other processes take place during the prenatal stage in rodents, such as apoptosis, migration, differentiation, or synaptogenesis, all of which are completed in the first postnatal weeks of life (Rice and Barone, 2000).

Several authors have reported a variety of effects due to dietary restriction during the prenatal stage on different parameters in the physiology of the ARC. Specifically, maternal undernutrition reduces the postnatal source of plasma leptin and alters POMC expression in males from P4 to P30 (Delahaye et al., 2008); alters NPY, AgRP, and ObRb mRNA levels in females at 5 months of age (Fraser et al., 2016); decreases the orexigenic response to ghrelin at 9 months of age (Jia et al., 2008) and increases c-fos-containing cells in adult males (Breton et al., 2009). However, to the best of our knowledge, there are no previous studies on the effects of the maternal diets low in proteins and calories (LPC) during gestation on the morphology of the ARC, nor on the differential effect that these severe dietary alterations may have on the ARC of the male and female offspring.

Given the number of processes that take place during the prenatal stage in rodents, it is essential to understand the impact of severe dietary restrictions on the structure and function of the ARC due to its crucial involvement in the regulation of energy metabolism. Furthermore, it is well-established that the outcome of the different processes that take place during the development of the hypothalamic circuits that regulate food intake are different in males and females (Asarian and Geary, 2013; Chowen et al., 2018). However, most of this work has been carried out in one or the other sex, so it is essential to study both males and females in order to understand in-depth how undernutrition during prenatal development can affect the neural networks that regulate energy metabolism in the short or long term. For this reason, our study has two main objectives: first, we will investigate how an LPC diet affects ARC morphology in rats. Specifically, the volume of the structure, number of neurons, neuronal density and apoptosis will be studied. And second, we will study if LPC diets affect differentially during the prenatal stage to male and female rats.

## Materials and Methods

### Animals

Wistar rats were maintained in an automatically controlled room programmed at a temperature of 22 ± 2°C under a 12 h light cycle and 12 h dark cycle (lights on at 08:00) with water *ad libitum*. Throughout the study, animal care and handling practices were approved by the Local Ethics Committees and were in accordance with the European Union Directive (2010/63/UE) and the Spanish Government Directive (R.D. 53/2013). For mating, a male was placed in a cage with two females for 5 days. In order not to disturb the pregnant mothers during this period and due to the difficulty on many occasions of detecting the exact day of conception, the third day was considered as the gestational day (G) 0 for all pregnant mothers. On gestational day 6 (G6), 6 pregnant rats were divided into two groups. Three pregnant rats were fed *ad libitum* with a control diet (20% casein, Panlab, Barcelona, Spain) and 3 pregnant rats were fed *ad libitum* with a low protein (8% casein) and 30% of a calorie-restricted diet (Panlab, Barcelona, Spain). The composition of the diets is shown in Table 1.

**TABLE 1:** Composition of control and low-protein and low-calorie diets.

	Control diet	Low-PC diet
Proteins (%)	19.55	8.1
Fat (%)	5	5
Carbohydrates (%)	55.1	38
Minerals and vitamins (%)	8	8
Cellulose (%)	6	37.5
Energy Content (Kcal/kg)	3438	2294
Energy from protein (Kcal/kg)	22.8	14.10
Energy from fat (Kcal/kg)	13.1	19.60
Energy from carbohydrates (Kcal/kg)	54.1	66.30

On the day of birth, the pups were sexed and sacrificed. The litters of each restricted mother were 14 pups (64% males and 35% females), 12 pups (41% males and 58% females), and 15 pups (53% males and 46% females). The *ad libitum* mothers had litters as follows: 15 pups (53% males and 46% females), 14 pups (64% males and 35% females), and 14 pups (64% males and 35% females). Each litter was weighted grouped by sex and nutritional condition, as shown in Table 2. The following groups were established by random selection among the pups of the same experimental condition: control male group (CM; *n* = 5), control female group (CF; *n* = 5), a group of males fed with low-protein and low-calorie diets (LPCM; *n* = 5), and a group of males fed with low-protein and low-calorie diets (LPCF; *n* = 5).

**TABLE 2:** Mean of the body weights (g) for each litter depending on their nutritional condition.

Low-PC diet		Control diet	
Males	Females	Males	Females
6.24 ± 0.1 g.	5.88 ± 0.1 g.	6.44 ± 0.1 g.	6.03 ± 0.1 g.
7.56 ± 0.1 g.	6.77 ± 0.1 g.	5.40 ± 0.1 g.	5.17 ± 0.1 g.
5.96 ± 0.1 g.	5.60 ± 0.1 g.	6.99 ± 0.1 g.	6.51 ± 0.1 g.
8.54 ± 0.1 g.	7.62 ± 0.1 g.	7.85 ± 0.1 g.	7.24 ± 0.1 g.

### Histological Procedure and Morphometrical Analysis

On the day of birth, all animals in both studies were sacrificed by decapitation, and the brains were removed and fixed in 4% paraformaldehyde for 18 days at 4°C. After that, they were placed in 30% sucrose for 3–5 days. The brains were frozen and coronally cut into 40 μm-thick slices through the ARC with a cryostat (Microm HM 500-O) at –17°C. Sections were stained with a 0.1% solution of cresyl violet (61123, FLUKA).

To study the volume in the ARC, photographs of every fourth section of the nucleus were taken with a 10× magnification, taken with the NIKON Digital Sight DS-Fi1 camera connected to the NIKON Eclipse 80i microscope with the Olympus CellA version 1.1.6 program. These photographs were analyzed using the ImageJ program version 2.0.0.0-rc-69/1,52n.

The limits of the ARC were established according to the Atlas of the Developing Rat Nervous System (Paxinos et al., 1994), distinguishing between the anterior (coronal plates P0-25 to P0-34) and posterior (coronal plates P0-35 to P0-41) parts of the nucleus following a rostro-caudal axis.

**FIGURE 1:**
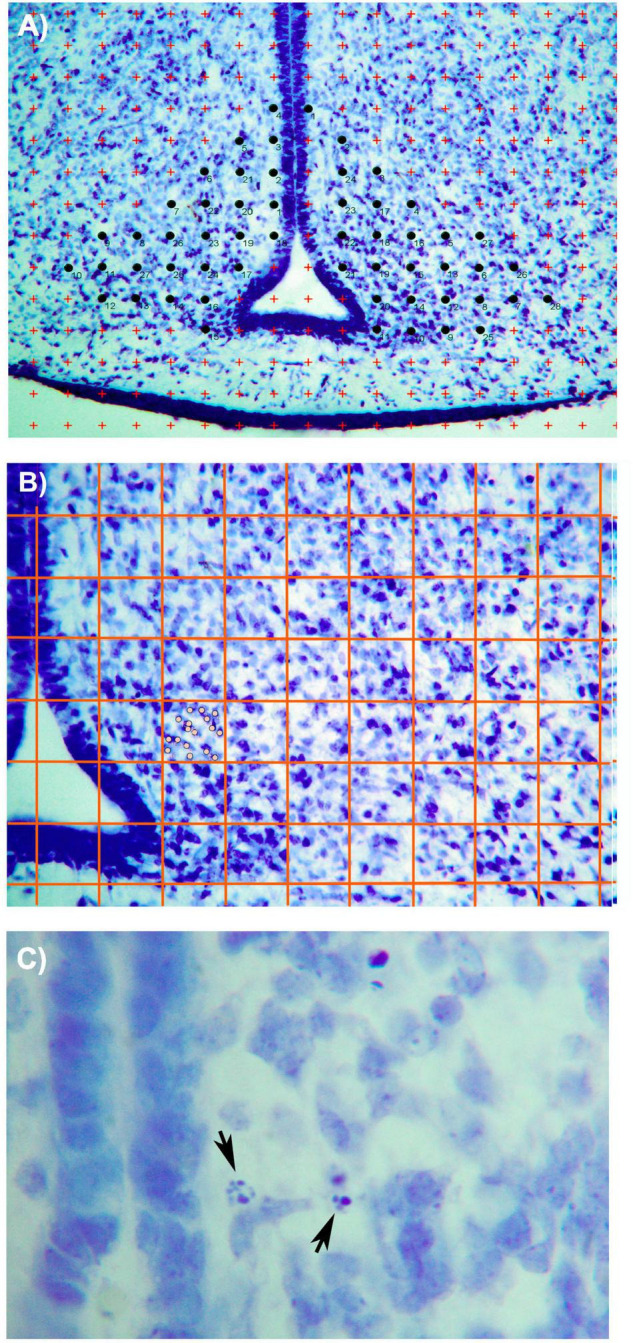
Photomicrographs (×10) showing **(A)** the Cavallieri method used for the estimation of ARC volume in Nissl-stained sections. Briefly, a grid of red crosses of 2,000 μ^2^ was overlaid on the photo. All crosses that are contained within the limits of the nucleus were counted (marked with black circles). **(B)** Method applied for counting neurons in the ARC section. A grid of red lines (these lines have been amplified in the photograph for an easier identification of the grid) was generated, covering all the sections. Each box has 0.04 μ^2^ size. As seen in the photo, all neurons (marked as gray circles) included within the limits of the box were considered. **(C)** Photomicrograph (×100) showing apoptotic activity in the ARC nucleus. Black arrows show two cells in different phases of the apoptotic process. Bar = 200 μm (Paxinos et al., 1994).

The right and left hemispheres were counted independently using a photograph taken with a 10× magnification. For the Cavalieri technique, a grid of crosses with an area per point of 2,000 μ^2^ was used (refer to Figure 1A). All the crossings of the grid located entirely within the limits of the ARC were counted in order to estimate the volume following Cavalieri’s formula. The researcher did not know the group to which the subjects she was evaluating belonged when measuring volume and the other parameters analyzed in this study.

To estimate the number of ARC cells, we used the same sections on which the volume was measured. A grid with 100 squares, with a 0.04 μ^2^ for each square, was placed on the microscope eyepiece. The grid was superimposed on the ARC image in each hemisphere, with the vertical line of the grid always aligned with the boundary of the third ventricle (refer to Figure 1B). Random numbers were used to determine the beginning of the squares to be counted. After counting the initial square, the cells of 1 out of every 25 squares were counted. Only cells included in the square were counted and those with part of the nucleus in the adjacent square were discarded.

To calculate the neuronal density, the number of estimated cells was divided by the volume of the nucleus. The number of pyknotic cells was determined in the same sections used to estimate the number of ARC neurons. We followed the criteria from Clarke’s (1990) study, which states that cell death by apoptosis begins with the condensation of both the nucleus and cytoplasm. Dense chromatin masses appear and increase in number in the nucleus until it becomes pyknotic condensed, which is the typical morphology of these dying cells (Figure 1C pyknotic).

In order to estimate the number of apoptotic cells in the ARC, a 25× magnification (NIKON Eclipse 80i microscope) was used. All cells in which the chromatin’s condensation was clearly distinguished were counted.

### Statistical Analysis

The volume, number of cells, neuronal population density, and apoptosis in the anterior and posterior subdivisions of the arcuate nuclei described by Paxinos et al. (1994) in both hemispheres were estimated. The data were submitted to one-way ANOVA, with the hemisphere as a factor to determine the potential differences between the right and left hemispheres. Once the effect of the hemisphere was discarded, the mean value of the two hemispheres and in each subdivision was used for the posterior statistical analysis. To determine sexual dimorphism, two-way ANOVA (with sex and diet as factors) was developed. Differences between groups were analyzed with S–N–K *post hoc* tests. The significance level was set at *p* < 0.05. To determine intrasex differences, male and female groups were analyzed independently using a one-way ANOVA (diet) with a significance level at *p* < 0.05. All values are expressed in terms of mean and SD, as shown in Table 3.

**TABLE 3:** Mean and SD values of morphological parameters studied.

	Volume	Anterior volume	Posterior volume	Cells	Anterior cells	Posterior cells	Neuronal density	Anterior neuronal density	Posterior neuronal density	Apoptotic cells
CM	0.0187 ± 0.0021	0.0116 ± 0.0027	0.0071 ± 0.0012	213.00 ± 28.69	137.90 ± 23.87	75.10 ± 9.21	5697.50 ± 578.09	6093.75 ± 1182.44	5492.60 ± 1478.40	6.80 ± 3.31
CF	0.0180 ± 0.0011	0.0103 ± 0.0025	0.0077 ± 0.0019	191.40 ± 55.11	108.20 ± 23.46	83.20 ± 33.61	5344.30 ± 1712.45	5489.82 ± 1656.83	5569.94 ± 2197.51	7.46 ± 4.33
LPCM	0.0190 ± 0.0014	0.0131 ± 0.0022	0.0059 ± 0.0011	267.70 ± 36.62	161.40 ± 32.84	106.30 ± 22.10	7042.40 ± 972.87	6164.39 ± 837.83	9075.32 ± 1515.76	6.66 ± 4.02
LPCF	0.0199 ± 0.0016	0.0116 ± 0.0022	0.0082 ± 0.0011	187.50 ± 29.18	104.90 ± 33.54	82.60 ± 26.15	4738.50 ± 846.87	4480.02 ± 1193.04	4993.24 ± 1249.57	4.66 ± 2.70

## Results

### Volume

Due to the moment of the development of the animals, there is a different cellular arrangement from the adult in the ARC. As shown in Figures 1A,B, the cells in P0 animals are densely packed, but the distinction of the cells can be made clearly with the Nissl stain. In addition, the delimitation of the ARC and their subdivision can be distinguished as explained in the section “Materials and Methods.”

Although a main effect of sex on the posterior ARC [*F*(1,16) = 5.370; *p* < 0.03] was found, *post hoc* analysis showed no significant differences among groups. When examining each sex separately, no differences were detected between female and male groups. Values of this parameter, expressed as mean and SD, as shown in Table 3.

### Number of Cells

**FIGURE 2:**
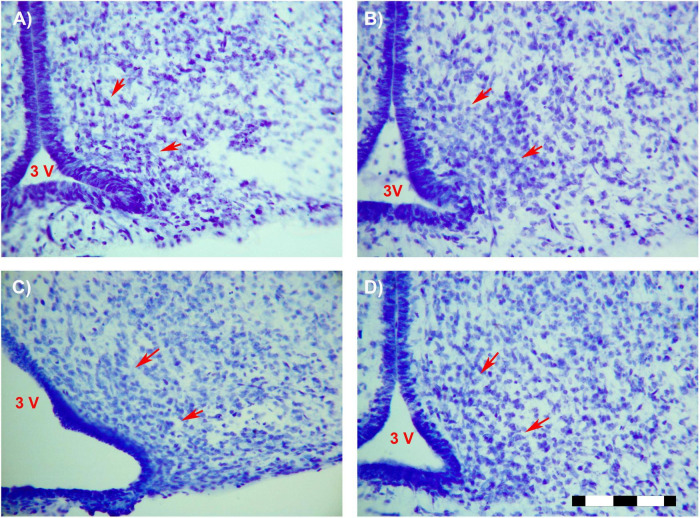
Photomicrographs (×25) showing the sexual dimorphism found between LPCM and LPCF. Limits of the ARC are signaled by red arrows. **(A)** CF; **(B)** CM; **(C)** LPCF; **(D)** LPCM. CM, control male; CF, control female; LPCM, low-protein and low caloric male; LPCF, low-protein and low caloric female. Bar = 100 μm (Paxinos et al., 1994).

**FIGURE 3:**
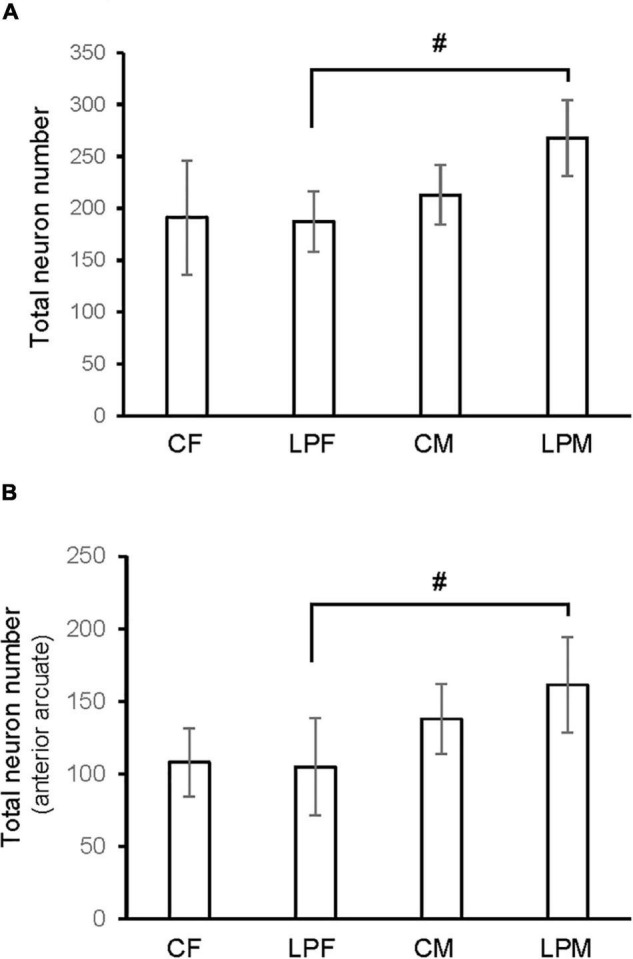
Histogram shows the differences in number of neurons in **(A)** ARC nucleus **(B)** anterior ARC subdivision. CM, control male; CF, control female; LPCM, low-protein and low caloric male; LPCF, low-protein and low caloric female. # significant differences between groups (*p* < 0.05 in all cases). All values are expressed as means ± SD.

A main effect of sex on the number of neurons in the total ARC [*F*(1,16) = 8.559; *p* < 0.01] and in the number of neurons of the anterior ARC [*F*(1,16) = 11.174; *p* < 0.004] were detected. *Post hoc* analysis showed the existence of sexual dimorphism between undernourished groups (LPCM vs. LPCF), having a higher number of neurons in males than females in the total ARC (*p* < 0.05) (Figures 2, 3A). When examining the subdivisions separately, we found statistically significant differences in the anterior subdivision in which LPCM > LPCF (*p* < 0.05) (Figure 3B).

**FIGURE 4:**
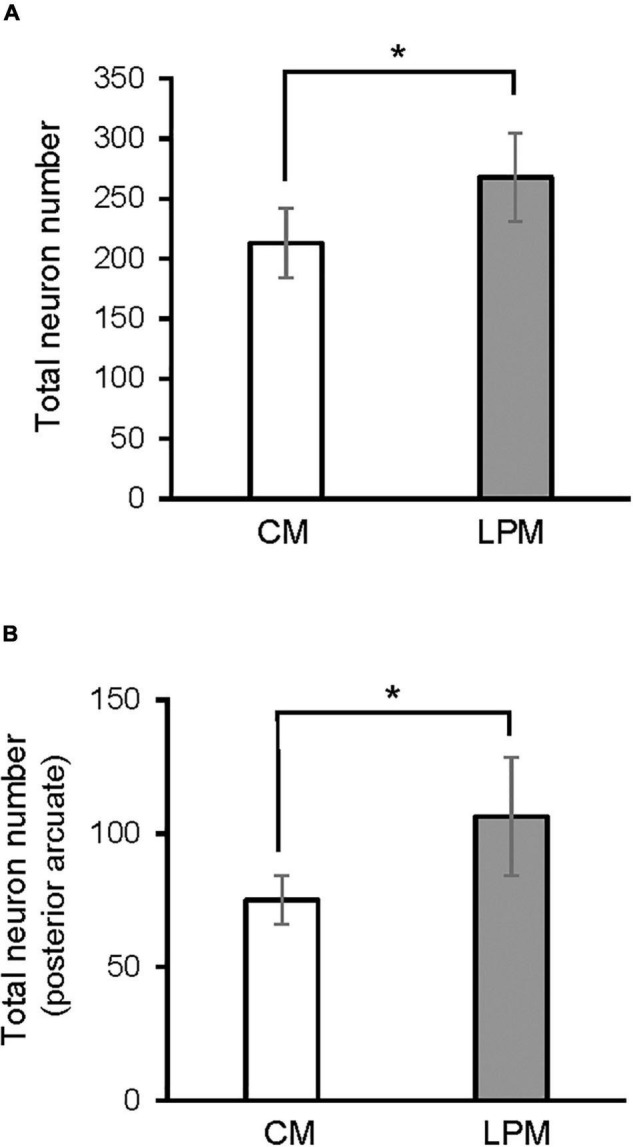
Graph shows the differences in number of neurons in **(A)** ARC nucleus **(B)** posterior ARC subdivision in males. CM, control male; LPCM, low-protein and low caloric male. *Significant differences between groups (*p* < 0.05 in all cases). All values are expressed as means ± SD.

**FIGURE 5:**
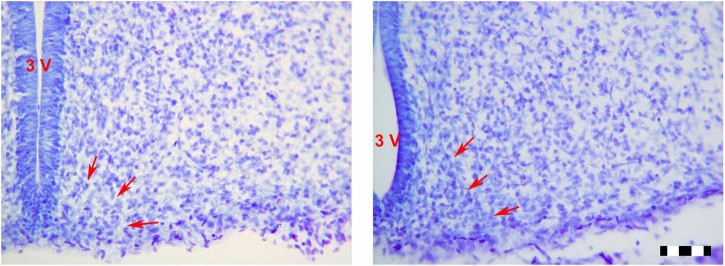
Photomicrographs (×25) showing differences between the male groups in posterior ARC. Limits of the ARC are signaling by red arrows. **(A)** CM; **(B)** LPCM. CM, control male; LPC, low-protein and low caloric male. Bar = 50 μm.

When analyzing each sex separately, no differences were detected between female groups. In males, a significant difference was detected between the two male groups, showing that LPCM has a greater number of neurons than CM in the total ARC [*F*(1,9) = 6,911; *p* < 0.03] (Figure 4A). This difference was due to an increase in the number of cells on the posterior subdivision in which a significant difference was found [*F*(1,9) = 8.487; *p* < 0.019] (Figures 4B, 5). All values are expressed as mean and SD, as shown in Table 3.

### Neuronal Population Density

**FIGURE 6:**
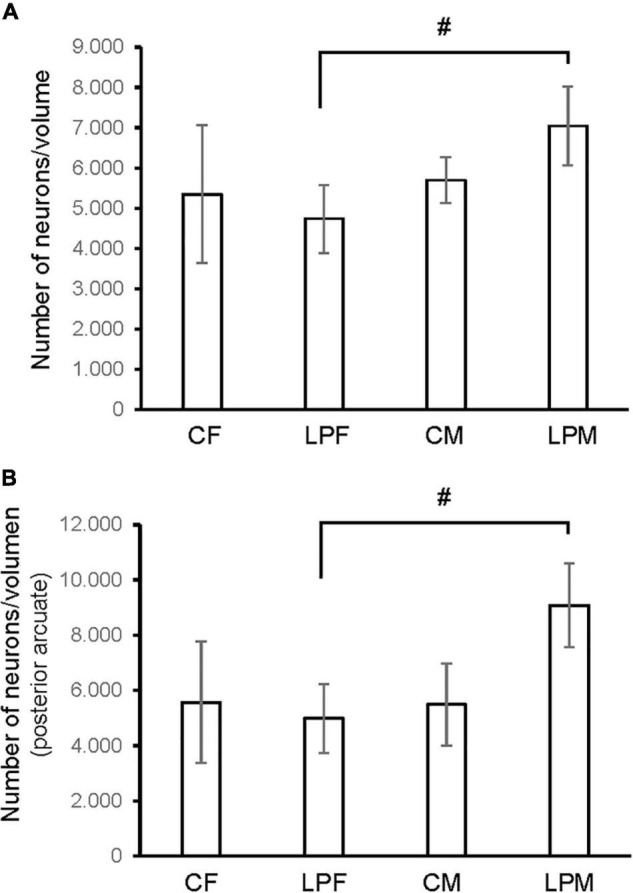
Histogram shows the differences in neuron density (calculated as number of neurons/volume) in **(A)** ARC nucleus **(B)** posterior ARC subdivision. CM, control male; CF, control female; LPCM, low-protein and low caloric male; LPCF, low-protein and low caloric female. # Significant differences between groups (*p* < 0.05 in all cases). All values are expressed as means ± SD.

**FIGURE 7:**
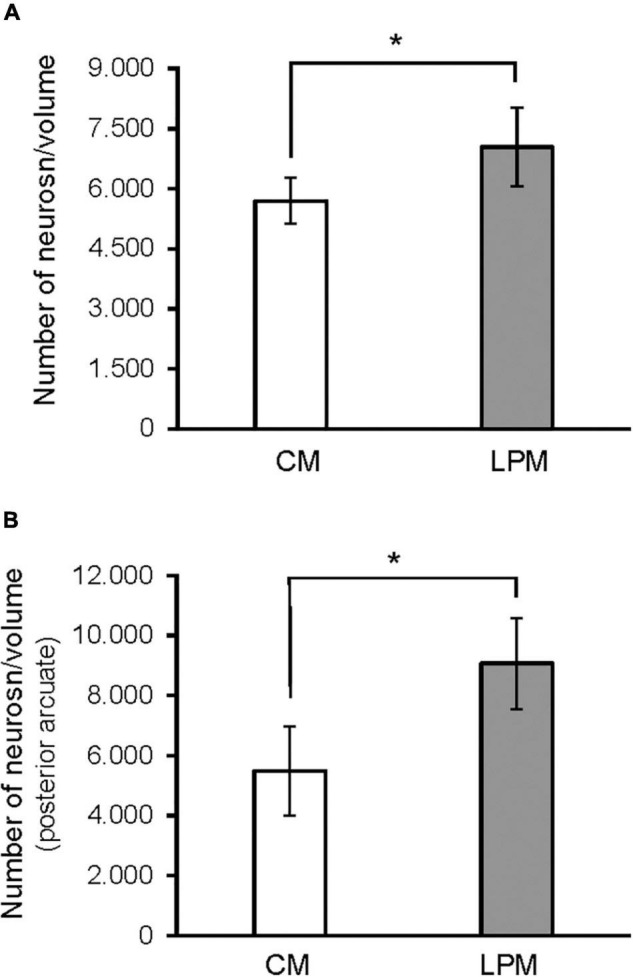
Graph shows the differences in neuron density (calculated as number of neurons/volume) in **(A)** ARC nucleus **(B)** posterior ARC subdivision in males. CM, control male; LPCM, low-protein and low caloric male. *, significant differences between groups (*p* < 0.05 in all cases). All values are expressed as means ± SD.

A main effect of sex on the neuronal population density in the total ARC [*F*(1,16) = 7.160; *p* < 0.01] and in the neuronal population density in anterior [*F*(1,16) = 4.177; *p* < 0.05] and posterior subdivisions [*F*(1,16) = 7.375; *p* < 0.01] was detected. A main effect of diet [*F*(1,16) = 4.15; *p* < 0.05] and the interaction [*F*(1,16) = 7.955; *p* < 0.012] on the posterior subdivision was also found. *Post hoc* analysis showed sexual dimorphism between LPCM and LPCF groups, with males showing a greater density of neurons than females (*p* < 0.05) (Figure 6A). When examining the subdivisions separately, we found statistically significant differences in the posterior subdivision of the ARC (Figure 6B), showing LPCM has higher neuronal population density than LPCF (*p* < 0.05) (Figures 7A,B). All values are expressed as mean and SD, as shown in Table 3.

### Apoptosis

Not many apoptotic cells were detected in the ARC, but the ones that were found were easily identified as given in Figure 1C. When analyzing this parameter, no significant effects of sex, diet, or the interaction between the two factors were found in this parameter. All values are expressed as mean and SD, as shown in Table 3.

## Discussion

The nutritional status of the mother during gestation largely determines the phenotype of the offspring since it is a well-known fact that severe dietary imbalances lead to a malfunctioning of the systems that control homeostasis, which mostly results in the onset of metabolic and eating disorders (Taylor and Poston, 2007; Desai et al., 2015; Rivera et al., 2019; Sertie et al., 2020). Our results confirm that not only the physiology, as other authors have reported, but also the morphology of the ARC is altered in newborn rats when they have been exposed to an inadequate nutritional environment in the prenatal stage.

The results of this study show that, on the day of birth, there are no differences between males and females in the volume, number of neurons or neuronal density of the ARC, or in any of its subdivisions in control animals. However, sexual dimorphism has been reported in some morphological and neurophysiological parameters of the ARC in adult rodents. In fact, it has been reported that both the volume and the number of neurons in the ARC were greater in males than females, but the number of synaptic spines, dendritic branching, and spine densities showed greater values in females than in males (Matsumoto and Arai, 1980; Leal et al., 1998). More recent works have also shown that males and females differ in the pattern of activation of vasopressin and vasoactive intestinal peptide on kisspeptin neurons of this structure (Schafer et al., 2018) and that the co-expression patterns of TH and nNOS with GABA neurons are also sexually dimorphic in the ARC in mice (Marshall et al., 2017).

In addition to adults, sex differences have also been reported in the ARC during the early stages of development. In this respect, it has been shown that astrocyte morphology is sexually dimorphic at birth. This pattern extends until adulthood (Mong et al., 1999; Mong and McCarthy, 2002). Moreover, the expression of GAD mRNA was higher in newborn males than in females (Davis et al., 1996), and the generation of TH neurons in females was more advanced than in females at E16 (Balan et al., 1996). However, there is no information about sex differences in volume or number of neurons in the ARC in young animals. Our results, coupled with the lack of sexual dimorphism in these parameters at birth, prove that the volume, number of neurons, and neuronal density of ARC are similar in both sexes. Since previous studies reported sex differences in these parameters in adult rats (Leal et al., 1998), the postnatal stage seems to be a crucial period for the development of the ARC, which makes it highly vulnerable to exposure to adverse external factors such as dietary restrictions. Our results showed that a prenatally low-protein and low-calorie diet can alter the balance shown by newborn control animals, given that sexual dimorphism appeared in the number of neurons and neuronal density in the LPCD groups. Those effects are mainly due to an increase in the number of neurons in LPCD males along with a slight decrease in this parameter in LPCD females. Interestingly, although the anterior subdivision showed sex differences in the number of neurons in these groups, it was the posterior subdivision that presented sex differences in the neuronal density in the LPCD groups. A combination of variations in volume and neurons in both subdivisions may contribute to the differential results of neuronal density in both subdivisions in restricted-fed diet animals.

Malnutrition during the perinatal period produces different alterations in the number of neurons in several brain structures. It has been reported that dietary restrictions between gestational day 16 and postnatal day 30 produced a decrease in the granule cells of the dentate gyrus of the hippocampus in male rats (Bedi, 1991). Low-protein diets during gestation and lactation produced an increase in neuronal density in the ventromedial and paraventricular nuclei of the hypothalamus (Plagemann et al., 2000), and the previous work by our research group has shown that malnutrition during the perinatal period produced a decrease in the neuronal population of the locus coeruleus in male and female rats at different postnatal ages from P7 to P60 days of age (Pinos et al., 2004, 2006).

However, other works have shown that malnutrition does not alter the neuronal population in other brain nuclei. Dietary restrictions during gestation do not produce changes in the neuronal population of the hippocampal pyramidal cells in CA2 and CA3 of male rats (Partadiredja and Bedi, 2010). Furthermore, low-protein diets during gestation and lactation did not lead to alterations in neuronal density in the lateral or dorsomedial hypothalamus (Plagemann et al., 2000).

The most significant result observed in our study is the large increase in the neuronal population in the ARC in newborn males prenatally exposed to a low-protein and low-calorie diet. These results may be surprising considering that, so far, all studies have shown that nutrient restriction either decreases (Bedi, 1991; Plagemann et al., 2000; Pinos et al., 2004, 2006) or does not affect the number of neurons in various structures, including the ARC (Plagemann et al., 2000; Partadiredja and Bedi, 2010). However, it must be noted that, in our study, the results of malnutrition have been analyzed at birth and not at later ages as in previous studies. At birth, many processes are still underway as the connections between the ARC and the other hypothalamic structures that control food intake do not acquire an adult pattern until about P16 (Bouret et al., 2004). Therefore, the structure at birth is still developing, thus not yet fully differentiated. What is more relevant to highlight is that the restriction in the diet during the gestational period can destabilize the normal development of the ARC in male offspring and that the consequence may be an alteration in the functioning of the structure that may lead to the development of eating disorders later in life. On the contrary, the fact that the LPC male group showed a significant increase in the number of neurons in the posterior subdivision but not in the anterior one might be due to the presence of different cellular types (Campbell et al., 2017) with different maturation timing in both subdivisions of the nucleus, which would reflect regional differences in the final adjustment of the neuronal population.

Most of the studies reported have been conducted on only one of the sexes, mainly males. But our results show that the data cannot be generalized since the two systems, males and females, do not respond in the same way to malnutrition, as has been previously shown. Studies carried out by our research group showed that males subjected to a high-fat diet from the second week of gestation decreased their body weight and their subcutaneous fat when analyzed as adults, but this did not occur in females, who maintained their body weight and fat depots like those of the control groups. However, in females, there was an increase in POMC mRNA levels that was not observed in males (Pinos et al., 2018; Carrillo et al., 2019). In addition to this, it was also observed that rats subjected to a prenatal caloric restriction presented a different response to nutritional rehabilitation if this occurred on postnatal day 12 or 21 (Pinos et al., 2004). All these results suggest that it is crucial to carry out research where males and females are used to understand the mechanisms that underlie the development of the hypothalamic circuits that regulate food intake in each sex. The reason why is that the fact that a low-protein and low-calorie diet does not have an effect on the volume or on the neuronal population of females does not mean that they are not affected. So it could be that the restriction of the diet produces some alterations in some other parameters not analyzed in this study and that may be identified later during their subsequent development or adulthood. For this reason, it is necessary to carry out more studies in order to gain an in-depth understanding of the altered mechanisms underlying prenatal diet restriction and of the periods, which are considered to be most susceptible to being altered due to unbalanced diets.

Regarding cell density, this parameter is the ratio between the number of neurons and the volume, which in the case of significant differences in any of the groups may suggest a greater or lesser cell dispersion or an alteration in volume or neurons individually. This last case is what has been observed in our study since the differences in neuronal density coincide with those found in the number of neurons. This means that, in this case, the differences in the ratio observed in males seem to reflect the increase in the number of neurons.

Concerning the mechanisms that may underlie the greater vulnerability of males to a prenatal low-protein and low-calorie diet, we have not been able to demonstrate that a lower rate of apoptosis could be the mechanism responsible for the increase of neurons in males in our research work. However, it does not mean that this mechanism is not involved since apoptosis occurs in a short period of time (Kerr et al., 1972) and what is observed at a specified time is the product of the rate of proliferation together with the death of neurons that have occurred in the previous hours/days. In addition to this, it is necessary to take into account that a wide variety of neuronal populations coexist in the ARC and that each neuronal phenotype can mature at a different time since they express different neurotransmitters and neuropeptides involved in diverse metabolic processes (Campbell et al., 2017). Therefore, more research is needed to find out which processes are altered by imbalances in the diet during the early stages of development and to determine which neuronal populations are most affected.

## Conclusion

The results of our work highlight, first of all, the importance of the diet during the prenatal stages. Besides, they have proved that a low-protein and low-calorie diet of the pregnant mother can alter the neuroanatomy of a structure that is so relevant to the control of food intake as the ARC. Secondly, they show that the neuronal population of this structure is vulnerable to a low-protein and low-calorie diet during the prenatal stage in males but not in females, which reinforces the idea of the need to study both sexes when the hypothalamic circuit that controls eating is analyzed. Only in this way will it be possible to identify in each sex the factors that jeopardize the maturation of the neural systems that regulate feeding. Knowing the mechanisms involved in the development of the circuits that regulate energy metabolism and feeding in males and females is essential to determine how different risk factors affect their development and, in the long run, it will allow us to promote strategies for the prevention and treatment of eating disorders.

## Data Availability Statement

The raw data supporting the conclusions of this article will be made available by the authors, without undue reservation.

## Ethics Statement

The animal study was reviewed and approved by the committees and were in accordance with the European Union Directive (2010/63/UE), Spanish Government Directive (R.D. 53/2013) and Comité de Bioética de la Universidad Nacional de Educación a Distancia.

## Author Contributions

PC, HP, and BC: conceptualization, formal analysis, writing—original draft, supervision, and review and editing. JF-G, NB, AB, and RG–U: methodology. PC and HP: project administration and funding acquisition. All authors has made substantial contributions to the work and approved the final manuscript.

## Conflict of Interest

The authors declare that the research was conducted in the absence of any commercial or financial relationships that could be construed as a potential conflict of interest.

## Publisher’s Note

All claims expressed in this article are solely those of the authors and do not necessarily represent those of their affiliated organizations, or those of the publisher, the editors and the reviewers. Any product that may be evaluated in this article, or claim that may be made by its manufacturer, is not guaranteed or endorsed by the publisher.
